# Functionalized Metal–Organic Frameworks Integrated with Plasmonic Nanoparticles: From Synthesis to Applications

**DOI:** 10.3390/bios16010053

**Published:** 2026-01-10

**Authors:** Songsong Huang, Qian Chen, Yanjun Li, Liyang Duan, Xuexing Zhao, Yanli Lu, Zetao Chen

**Affiliations:** 1 School of Disaster and Emergency Medicine, Tianjin University, Tianjin 300072, China; 2 Wenzhou Safety (Emergency) Institute of Tianjin University, Wenzhou 325026, China

**Keywords:** plasmonic nanoparticles, metal–organic frameworks, synergistic effect, surface enrichment, biomedical applications

## Abstract

Plasmonic nanoparticles (NPs) exhibit exceptional optical and electromagnetic (EM) properties that are, however, confined to their near–field region, limiting effective interactions with non-adsorbed species. Metal–organic frameworks (MOFs), renowned for their high surface area and tunable pores, provide an ideal complement through surface enrichment and subsequent molecular enrichment within their pores. The integration of plasmonic NPs with MOFs into nanohybrids overcomes this spatial constraint. This architectural synergy creates a synergistic effect, yielding properties superior to either component alone. This review summarizes recent advances in NP–MOF nanohybrids, with a focus on synthesis strategies for diverse architectures and their emergent functionalities. We highlight how this synergistic effect enables breakthrough applications in chemical sensing, cancer therapy, and catalysis. Finally, we conclude our discussion and present a critical outlook that explores the challenges and future opportunities in the design and applications of NP–MOF nanohybrids.

## 1. Introduction

Traditional chemical and biological sensing technologies face numerous challenges in practical applications. First, they often suffer from insufficient sensitivity and are prone to background interference in complex media, making it difficult to achieve trace-molecule detection [1]. Second, their reliance on labeling makes the operational process cumbersome and incurs additional costs and may alter the intrinsic properties of the target analytes [2]. Third, real-time monitoring of dynamic molecular interactions remains challenging, limiting applications in scenarios requiring rapid responses [3].

The localized surface plasmon resonance (LSPR) effect of noble metal plasmonic NPs (particularly Au, Ag, and Cu) provides an effective solution to overcome these challenges [4,5,6]. Although the scope of plasmonic materials has expanded to include non-metallic systems (e.g., nitrides like TiN and ZrN), noble metals remain the most prominent due to their ability to support strong and tunable LSPR in the visible region. Consequently, this review will primarily focus on noble metal plasmonic NPs, and the term “plasmonic NPs” in the following text specifically refers to these materials unless otherwise stated. These plasmonic NPs exhibit two distinct optical characteristics derived from LSPR: intense absorption and scattering of light, as well as a pronounced enhancement of the EM field due to light–matter coupling. The shift in the LSPR extinction peak offers a direct, label-free sensing mechanism [7,8]. Additionally, the efficient light-to-heat conversion of plasmonic NPs creates highly localized thermal zones around the particles, enabling photothermal approaches in biomedical fields such as cancer treatment [9,10]. Meanwhile, the concentrated EM fields at the surfaces of plasmonic NPs form the basis of surface-enhanced spectroscopic techniques, including surface-enhanced Raman spectroscopy (SERS) and surface-enhanced infrared absorption (SEIRA). These techniques amplify the vibrational signals—either Raman scattering or IR absorption—of molecules located within the region of enhanced EM fields [11]. As a result, plasmonic NPs can dramatically boost the otherwise weak molecular vibrational fingerprints [12]. Moreover, under laser illumination, reactions taking place near plasmonic surfaces are promoted through the transfer of hot carriers to adsorbed species, facilitating catalytic processes [13].

However, plasmonic platforms face a core bottleneck: their inefficiency in interacting with non-adsorbed or weakly adsorbed molecules. This is because the EM field enhancement associated with the LSPR effect decays exponentially with distance, preventing molecules far from the plasmonic surface from effectively utilizing the field enhancement characteristics [14]. Consequently, for low-concentration analytes or in non-specific adsorption systems, the lack of surface affinity hinders target molecule binding to the plasmonic surface, which prevents the effective exploitation of LSPR enhancement, severely limiting the practical applicability of plasmonic platforms.

**Figure 1:**
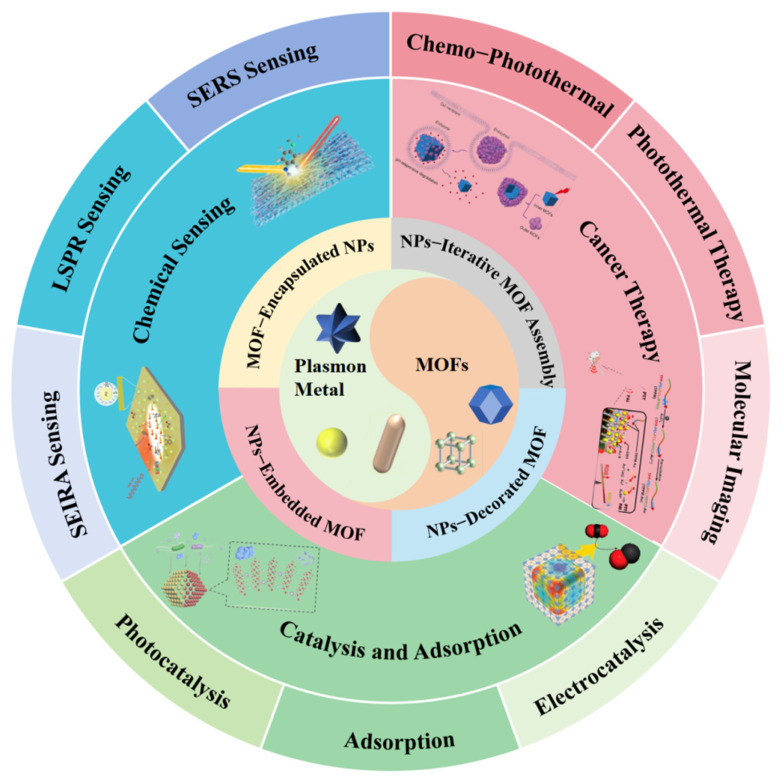
Overview of the emerging configurations and applications of plasmonic NP–MOF nanohybrid platforms. Reprinted and adapted with permission from references [15,26,27,28,29,30]. Copyright 2016 and 2024 American Chemical Society. Copyright 2017 Ivyspring International Publisher.

To address this spatial limitation, researchers have attempted to combine adsorbent materials with plasmonic NPs. Through this hybrid design, analytes can be preconcentrated in the near-field region of plasmonic NPs, which enhances their interaction with the local EM field, thereby achieving the goals of signal amplification and improved catalytic performance [15]. Among adsorbent materials, MOFs, as a class of three-dimensional porous crystals formed by the coordination of metal ions and organic linkers, possess a series of excellent properties. First, the specific surface area of MOFs typically ranges from 1000 to 10,000 m^2^/g, far higher than that of traditional porous materials such as silica, zeolites, and polymers [16,17]. Second, by selecting different metal ions, organic ligands (e.g., carboxylate-based, phosphonate-based, and sulfonate-based ligands), and preparation conditions (e.g., temperature, concentration, pH), the pore structure, chemical properties (e.g., acidity–alkalinity, hydrophilicity–hydrophobicity), and functions (e.g., catalytic activity, adsorption selectivity, proton conductivity) of MOFs can be precisely tailored to meet specific needs [18,19]. Third, over 90,000 varieties of MOFs have been synthesized, with estimates suggesting the potential existence of more than 500,000 structures [20,21]. Currently, artificial intelligence (AI) is being employed to predict novel MOF syntheses, further expanding this diversity. In contrast, traditional porous materials often have relatively rigid pore sizes and limited micropore variety, constraining their adsorption range [22]. Consequently, integrating plasmonic NPs with MOFs has garnered significant interest. This system synergistically combines the strengths of both components, offering distinct advantages over using plasmonic NPs alone. MOFs excel at adsorbing and preconcentrating analytes, including gases and vapors [23,24]. In addition, their tunable pore sizes enable a molecular sieving effect, selectively admitting target analytes while excluding larger interfering molecules. MOFs can also act as a protective shell around NPs, effectively preventing their aggregation and significantly enhancing their chemical stability. This integration also gives rise to synergistic effects; for example, the charge transfer between plasmonic NPs and MOFs can potentially improve photocatalytic efficiency, while the tailored pore environment of MOFs can enhance selective adsorption for separation applications and gas storage [25]. Thus, the combination of NP–MOF mitigates individual drawbacks while amplifying inherent advantages, propelling advancements in chemical sensing, cancer therapy, catalysis, and adsorption (Figure 1).

Despite notable advances in plasmonic NP–MOF hybrid platforms, a systematic overview that synthesizes recent progress remains scarce. Such a consolidation is essential to map future research directions. Recognizing the multifunctional benefits and adaptability of these nanohybrids, this review aims to assess the current landscape critically, showcasing key breakthroughs while pinpointing existing knowledge gaps—thereby accelerating the further development and application of plasmonic NP–MOF systems. This review begins with a systematic overview and contrast of the preparation strategies for hybrid materials comprising plasmonic NPs and MOFs. Subsequently, the discourse will focus on the distinctive synergistic effects emerging from the integration of these components.

The application spectrum of these hybrid materials will be extensively explored, covering advances in multiple domains: chemical sensing (utilizing techniques such as LSPR, SERS, and SEIRA), cancer therapy (including combination chemo-photothermal therapy, photothermal therapy, and molecular imaging), as well as catalysis and adsorption processes (with a focus on electrocatalysis, photocatalysis, and adsorption-based applications) [31,32,33,34,35]. Finally, we will conclude our discussion and present a critical outlook that explores the challenges and future opportunities in the design and applications of NP–MOF nanohybrids. We hope that these insights can promote the further development of the plasmonic and MOF fields and bring new solutions to challenges in environmental science, industrial processes, biomedicine, and chemistry.

## 2. Types of Np–Mof Platforms

### 2.1. MOF–Encapsulated NPs

Growing MOFs on the surface of plasmonic NPs is a common method for constructing hybrid materials. Generally, the plasmonic NPs are synthesized first, followed by the nucleation and growth of MOFs to fully encapsulate the plasmonic core. This process is typically carried out in solution, as the solution environment facilitates better preservation and regulation of the morphologies of both plasmonic NPs and MOFs [36]. The advantage of this method is that NP–MOF composites with controllable optical responses can be obtained by adjusting the size and morphology of the plasmonic NPs. In this process, overcoming the interfacial energy barrier between NPs and MOFs and achieving heterogeneous nucleation and growth are critical for successful MOF formation. However, the inherent lattice mismatch between plasmonic NPs and MOFs results in high interfacial energy, which typically inhibits heterogeneous nucleation and growth on the metal surface, posing a significant challenge in encapsulating plasmonic NPs with MOFs.

Early studies proposed a strategy for depositing MOFs onto plasmonic NP surfaces via electrostatic interactions. For example, heterogeneous nucleation of MOF crystals has been successfully guided by leveraging the electrostatic attraction between their metal nodes and the surface charges of functionalized Au NPs [37]. This approach was further optimized by promoting complete contact between Pd NPs and MOF precursors through ultrasonic dispersion. This process facilitated the electrostatic adsorption of ions and ligands on the Pd NP surface, thereby guiding MOF nucleation [38]. However, encapsulation relying solely on electrostatic forces has limitations. First, the weak electrostatic binding strength makes it difficult to stabilize the nucleation sites on high-curvature surfaces, leading to random MOF growth directions. Second, dynamic changes in solution ion concentration during MOF growth can exacerbate Ostwald ripening of the plasmonic NPs, leading to uneven encapsulation layers, increased porosity, and local thickness variations [39]. Such non-uniform coatings weaken the synergistic effect between the plasmonic near-field and MOF pores, compromising the composite’s performance in catalysis and sensing. To overcome these limitations and achieve more controlled encapsulation, two primary strategies have emerged: modifying plasmonic NPs with surfactants and functionalizing NP surfaces with a sacrificial template.

#### 2.1.1. Surface Modifier Control Strategy

To overcome the limitations of electrostatic interactions, a surfactant-mediated strategy for encapsulating NPs within MOFs was developed. Key surfactants include polyvinylpyrrolidone (PVP), cetyltrimethylammonium bromide (CTAB), and thiolated polyethylene glycol (PEG–SH), each with distinct regulatory functions:

(1) Role of PVP

The PVP molecule contains polar amide groups and nonpolar alkyl chains. The polar groups (C=O in the pyrrolidone) can form weak coordination interactions with NP surfaces and MOF metal nodes, anchoring NPs and guiding MOF nucleation. The nonpolar segments bind to MOF organic ligands via hydrophobic interactions, precisely regulating MOF growth position on the NP substrate and optimizing NP spatial distribution [40,41]. It should be noted that the PVP concentration is critical: too low leads to NP aggregation; too high can cause MOF structural defects [42]. This strategy enables precise encapsulation of diverse plasmonic NP shapes within MOFs as core templates, significantly enhancing the structural tunability of NP–MOF composites.

**Figure 2:**
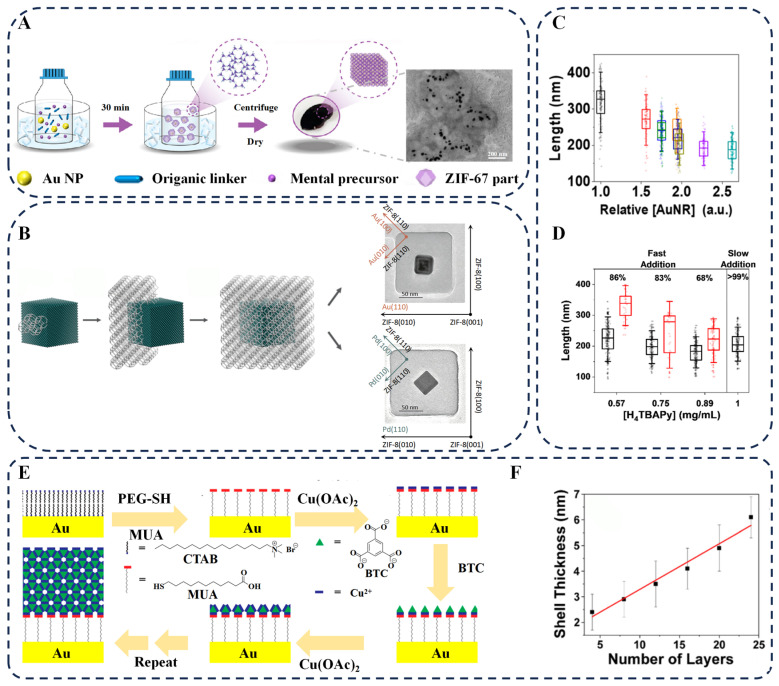
(**A**) Synthesis procedure of core–shell Au@ZIF-67 nanostructures. (**B**) Growth mechanism of aligned NP@ZIF-8: surfactant-guided oriented attachment followed by epitaxial shell growth. (**C**) Size statistics of Au NR@NU-901 synthesized with varied Au NR concentrations. (**D**) Size statistics and encapsulation yield of Au NR@NU-901 synthesized with varied ligand amounts. (**E**) Synthetic conditions for Au NRs coated with HKUST–1 through NP-mediated iterative MOF assembly. (**F**) HKUST–1 shell thickness from TEM analysis plotted vs. the number of layers deposited. Reprinted and adapted with permission from (**A**) reference [44], (**B**) reference [45], (**C**,**D**) reference [46], and (**E**,**F**) reference [47]. Copyright 2014, 2018, and 2019 American Chemical Society. Copyright 2022 ELSEVIER.

The research by Li et al. also demonstrates this mechanism: the carbonyl group of PVP coordinates with Zr^4+^ to guide the growth of NH_2_–UiO–66 on the surface of Au NPs [43]. PVP not only sterically hinders Au NP aggregation but also coordinates to guide the ordered growth of CdS NPs on the MOF surface. The resulting Au@NH_2_–UiO–66/CdS composite exhibited excellent photocatalytic H_2_ production. Tian et al. expanded this application, utilizing weak coordination between PVP and ZIF–67 precursor Co^2+^ to make Au NPs ideal sites for ZIF–67 heterogeneous nucleation [44]. PVP sterically inhibited particle aggregation during growth, resulting in Au NPs uniformly embedded within ZIF–67 polyhedra, suitable for synergistic plasmonic and MOF confinement effects (Figure 2A).

It is noteworthy that such surfactant-mediated growth, where long-chain PVP molecules are unevenly distributed at the NP–MOF interface, often disrupts the isotropic crystal growth of MOFs. This disruption typically yields polycrystalline MOF shells with randomly oriented pores. While this structure effectively immobilizes and protects plasmonic NPs, it introduces distinct performance trade-offs: (1) For mass transport, the random pore orientation and inherent grain boundaries create more tortuous diffusion pathways, which increases mass transfer resistance and limits the accessibility of reactant molecules to the encapsulated catalytic NPs, which can lower the overall reaction rate and efficiency in catalytic applications. (2) For optical properties, the polycrystalline, granular texture of the shell enhances light scattering; this scattering not only attenuates the incident light intensity reaching plasmonic NPs but also disrupts the uniformity of the localized EM field generated around them—a critical factor for surface-enhanced spectroscopies and plasmon-driven catalysis, potentially leading to reduced enhancement factors and signal reproducibility in SERS sensing.

(2) Role of CTAB

The cationic head of CTAB can be electrostatically adsorbed onto the negatively charged surface of plasmonic NPs. Its nonpolar long-chain tail is hydrophobic and can interact with the hydrophobic regions of the MOF organic ligands through hydrophobic interactions. This effectively provides “aggregation points” for the attachment of MOF crystal nuclei and promotes their formation on the NP surface [48,49]. CTAB micelles act as microreactors: hydrophobic cores enrich hydrophobic ligands, while cationic shells electrostatically repel to inhibit anisotropic MOF growth, yielding uniform coatings with consistent thickness and low variation. This ability to direct and homogenize MOF growth makes CTAB-mediated assembly a powerful strategy for constructing well-defined NP@MOF core–shell structures.

For example, Wang et al. utilized CTAB-mediated interfacial assembly to achieve directional growth of ZIF–67 on the surface of silver nanowires (Ag NWs) [50]. By adjusting the precursor soaking time, the thickness of the ZIF–67 layer can be precisely controlled. The observed red shift in the plasmon resonance peak exhibits a linear correlation with the layer thickness, demonstrating the precise tunability of CTAB over the plasmonic properties of the system. Hu et al. further extended this strategy to the ZIF–8 system, achieving a well-defined 1:1 core–shell structure where single plasmonic nanocrystals are individually encapsulated in single-crystalline ZIF–8 matrices (Figure 2B) [45]. Notably, CTAB induces specific lattice alignment between the metal core and MOF shell—with the {100} planes of the metal nanocrystals aligning with the {110} planes of ZIF–8—while enabling continuous tunability of the ZIF–8 shell thickness from ~35 to 60 nm by fine-tuning the CTAB dosage. This strategy is highly versatile, applicable to plasmonic NPs with diverse shapes (cubic, octahedral) and compositions (Pd, Au), and can even be extended to direct the oriented overgrowth of mesostructured silica on the NP@ZIF–8 core–shell composites, further expanding its scope of application. The combination of CTAB with other molecules, such as tris(hydroxymethyl)aminomethane (TRIS), can also regulate the morphology of MOFs, greatly expanding the applicability of this approach [51].

(3) Role of PEG–SH

PEG–SH can be anchored to the surface of plasmonic NPs via the thiol group (–SH). Its hydrophilic ethoxy chains (–CH_2_CH_2_O–) form hydrogen bonds and dipole interactions with MOF precursors, serving as a key regulator for NP stability and in situ MOF nucleation [52]. Benefiting from its dual functions of stable anchoring and interfacial regulation, PEG–SH is a reliable choice for constructing precisely controlled NP@MOF architectures.

Osterrieth et al. modified Au nanorods (Au NRs) with PEG–SH: its long-chain steric hindrance effectively inhibits AuNR aggregation in harsh environments, while oxygen atoms in PEG interact with Zr^4+^ and H_4_TBAPy to induce preferential nucleation of NU–901 on Au NRs [46]. Acting as “seeds”, the concentration of PEG@Au NRs precisely tunes the MOF shell size (Figure 2C). With slow ligand addition, the core–shell encapsulation efficiency exceeds 99% and NU–1000 impurities are suppressed, significantly enhancing the composite’s selective SERS detection performance (Figure 2D).

#### 2.1.2. NP Surface Functionalization with a Sacrificial Template

Metal oxides (e.g., Cu_2_O, ZnO, Al_2_O_3_) are widely used to reduce the interfacial energy between plasmonic NPs and MOFs and promote the oriented growth of MOFs due to their tunable solubility and metal ion release capabilities. This strategy not only enhances chemical stability but can also enable the in situ release of metal ions to participate in MOF construction. Cu_2_O is one of the most commonly used sacrificial templates, often employed to coat Au NPs or Ag NPs to subsequently template MOFs like ZIF–8 [53,54]. In the presence of ZIF–8 precursors, deprotonation of 2–methylimidazole releases H^+^, lowering the local pH and etching the Cu_2_O shell. Subsequently, the addition of NH_4_OH completely removes the residual Cu_2_O, ultimately forming the NP@ZIF–8 structure, where the cavity size is precisely controlled by the thickness of the Cu_2_O shell. This core–shell structure not only retains the optical activity of plasmonic NPs but also endows the composite with the molecular sieve function of the MOF.

Al_2_O_3_ can also serve as a sacrificial template for MOF growth [55]. In an acidic medium, Al_2_O_3_ slowly dissolves to release Al^3+^ ions, which coordinate with 2–aminoterephthalic acid (H_2_BDC–NH_2_) to form MIL–53 (Al) [56]. This process involves four stages: the hydration of Al_2_O_3_ to form aluminum–hydroxyl species (AlOOH), the neutralization of AlOOH with H^+^ to release Al^3+^, the coordination of Al^3+^ with BDC^2–^–NH_2_ to form MIL–53 (Al) nuclei, and finally the growth of the nuclei into MOF crystals. The entire growth kinetics is regulated by pH, which promotes the release of Al^3+^ and inhibits the deprotonation of H_2_BDC–NH_2_. In addition, this strategy has also been used to encapsulate novel plasmonic materials, such as semiconductor NPs like Cu_2−x_Se or indium tin oxide (ITO) in ZIF–8, preserving their plasmonic properties and stability [57]. Furthermore, it has also been successfully applied to embed plasmonic oxides, such as molybdenum oxide (MoO_3_) [58]. Compared with noble metals, these oxides have higher photostability and lower cost, providing a new pathway for constructing efficient and stable NP–MOF composites.

In summary, the MOF-encapsulated NP method effectively overcomes high interfacial energy barriers, enabling precise positioning, uniform encapsulation, and functional integration of plasmonic NPs within the MOF matrix. These strategies significantly enhance the controllability and functionality of the core–shell structure, offering a versatile and efficient solution for designing high-performance NP–MOF composites. An overview of the types, sizes, and applications of MOF-encapsulated NPs is provided in Table 1. However, the success of this approach critically depends on the interaction strength between the modifier and the MOF precursor. Insufficient binding strength can lead to uneven NP distribution, incomplete encapsulation, or Ostwald ripening, as the MOF precursors fail to be effectively anchored to the NP surface for directed heterogeneous nucleation. Conversely, the use of strong but static binding modifiers introduces a different set of challenges. These molecules can become permanently trapped at the interface, creating an insulating amorphous layer that hampers charge transfer and physically blocks MOF pores, thereby negating the intended synergistic effects. Therefore, future efforts should focus on exploring more robust and universal interfacial engineering strategies that transcend simple physical adsorption. Promising directions include the following: (1) exploiting the dynamic nature of weakly adsorbed capping agents that can be in situ replaced by the growing MOF, resulting in a clean, aligned, and direct NP–MOF interface with superior catalytic selectivity [59]. (2) Designing chemospecific interactions, such as introducing dynamic covalent linking units on MOF surfaces, which allow for directional, modular, and intimate assembly with plasmonic NPs while minimally affecting the intrinsic porosity of the MOF [60].

### 2.2. NP-Mediated Iterative MOF Assembly

The NP-mediated iterative MOF assembly method enables precise, layer-by-layer deposition of MOF shells. Growth is driven by either electrostatic adsorption or coordination bonding between the NP surface and MOF precursors. Experimentally, this is typically achieved through one of two cyclic immersion approaches: (1) the repeated centrifugation and alternate immersion of plasmonic NPs themselves in precursor solutions, or (2) the cyclic immersion of NP-functionalized colloidal substrates into precursor solutions. Compared to direct one-pot encapsulation, this iterative approach allows precise control over shell thickness by varying the number of cycles, effectively suppresses nonspecific nucleation, and is highly compatible with anisotropic nanostructures [62,63].

#### 2.2.1. Construction of Core–Shell Structure by Liquid-Phase Centrifugal Circulation Method

When plasmonic NPs are dispersed in a solution, the NP-mediated iterative MOF assembly method achieves in situ growth of MOF films through cyclic centrifugation and alternate immersion. Hinman et al. modified Au NRs with 11–mercaptoundecanoic acid (MUA) and then alternately immersed them in Cu(OAc)_2_ and H_3_TMA–BTC solutions to initiate coordination-directed growth (Figure 2E) [47]. The carboxyl groups of MUA coordinate with Cu^2+^ to form initial nucleation sites, while H_3_TMA–BTC guides epitaxial MOF growth through coordination of its carboxyl groups with Cu^2+^. Each additional cycle linearly increases the MOF shell thickness by 0.178 nm. After 30 cycles, a fully encapsulated core–shell structure was obtained (Figure 2F).

This study demonstrates the advantage of the NP-mediated iterative MOF assembly method in achieving nanoscale-precision shell control and provides a general solution for the controllable encapsulation of MOFs on anisotropic nanomaterials.

#### 2.2.2. Preparation of Functional Films by Alternate Immersion for Substrate-Immobilized NPs

When plasmonic NPs are immobilized on a substrate, the NP-mediated iterative MOF assembly method enables in situ growth of MOF thin films through alternating immersion. He et al. chemically immobilized Au NPs via Au–S bonds, enhanced the interfacial binding strength through MUA modification, and then induced MOF growth by alternately immersing the substrate in Cu(OAc)_2_ and H_3_BTC solutions [64]. As the number of cycles increased from 40 to 120, the MOF structure evolved from a monolayer to dense, pyramid-shaped multilayers. This significantly improved the film’s detection performance for volatile organic compounds (VOCs), while exhibiting excellent reversibility and resistance to temperature and humidity interference. Molecular-level design of interfacial binding sites enables precise MOF growth on immobilized NPs.

This iterative assembly strategy represents a paradigm shift in NP–MOF encapsulation, moving beyond simple physical mixing to precise interfacial engineering. It fundamentally changes the fabrication paradigm by enabling programmable, layer-by-layer construction of the core–shell architecture. By controlling the number of deposition cycles, this method achieves nanoscale regulation over MOF shell thickness, uniformity, and interfacial properties, providing a versatile platform for designing functionally tailored core–shell structures. However, the primary drawbacks of this approach are its inherent complexity and low throughput. The process requires not only multiple cycles but also a finely tuned balance at each step to ensure heterogeneous nucleation on the surface while suppressing competing homogeneous nucleation in the solution. This necessitates meticulous optimization of parameters such as precursor concentration, immersion time, and intermediate washing, which collectively hinder scalability and reproducibility.

### 2.3. NP-Embedded MOF

A conceptually distinct and powerful strategy is the in situ synthesis of plasmonic NPs within pre-formed MOF hosts. This NP-embedded MOF approach typically involves impregnating the MOF pores with a metal precursor solution, followed by controlled reduction directly inside the confined space. This “ship-in-a-bottle” methodology effectively inhibits NP aggregation while maintaining MOF structural integrity. More importantly, it leverages the MOF’s pore chemical environment to regulate NP nucleation and growth at the molecular level. This is particularly advantageous because the pore sizes of many MOFs are smaller than typical pre-synthesized plasmonic NPs, making direct encapsulation challenging [65]. The essence of this in situ strategy is the use of the MOF not merely as a passive scaffold, but as an active, nanoreactor that templates the formation of well-defined NPs.

**Figure 3:**
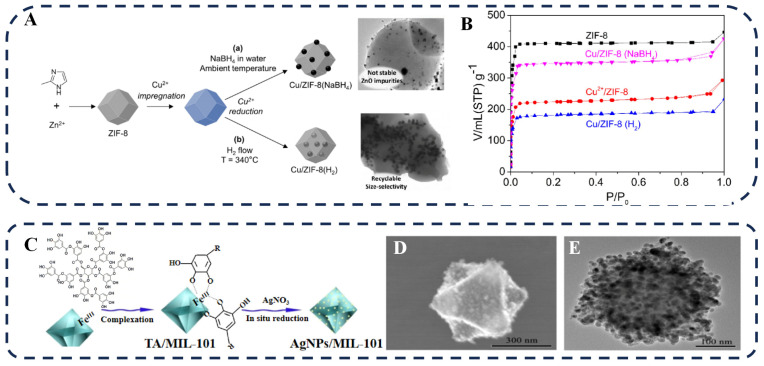
(**A**) Schematic illustration of Cu/ZIF–8 nanocomposite formation via two Cu^2+^ reduction methods: (a) NaBH_4_ reduction and (b) H_2_ reduction. (**B**) N_2_ adsorption isotherms of ZIF–8, Cu^2+^/ZIF–8, Cu/ZIF–8 (NaBH_4_), and Cu/ZIF–8 (H_2_). (**C**) Schematic of fabrication of Au NP-decorated MIL–101. SEM (**D**) and TEM (**E**) images of Ag NPs/MIL–101. Reprinted and adapted with permission from (**A**,**B**) reference [66] and (**C**–**E**) reference [67]. Copyright 2015 and 2023 American Chemical Society.

In contrast, the alternative approach—first introducing a metal precursor and then reducing it within the pores—can fully utilize the confinement effect of the MOF. This pore-confinement strategy has been successfully demonstrated in many systems. For example, Zan et al. utilized the pore structure of ZIF–8 and the coordination interaction between imidazolate ligands and Cu^2+^, enabling Cu^2+^ adsorption within the pores. They reduced Cu^2+^ through two methods: reduction under H_2_ at 340 °C to generate Cu NPs embedded inside the ZIF–8 crystals and reduction with NaBH_4_ solution to form Cu NPs distributed on the surface of ZIF–8 (Figure 3A) [66]. N_2_ adsorption–desorption isotherms confirmed that Cu/ZIF–8 (H_2_) had a lower specific surface area and pore volume than Cu/ZIF–8 (NaBH_4_), which is consistent with Cu NPs being predominantly embedded within the ZIF–8 pores in the former case, as opposed to being localized on the external surface in the latter (Figure 3B). The pore confinement effect ensured uniform dispersion and size stability of the embedded Cu NPs during catalytic cycles, preventing aggregation and loss, thus conferring higher catalytic stability compared to the surface-loaded counterpart.

In addition to external reducing agents, certain MOFs with redox activity can also serve as intrinsic reductants. Li et al. utilized redox-active MOFs based on Zr_6_ clusters and triphenylamine linkers. Through in situ redox between triphenylamine groups and Pd^2+^, Pd^2+^ was reduced to ultra-small Pd NPs within the MOF pores [68]. The pore confinement effect of MOFs effectively prevented Pd NP aggregation. The composite material exhibited excellent activity and good reusability in the Suzuki–Miyaura coupling reaction.

The NP-embedded MOF method is ideal for applications requiring small, highly dispersed NPs. An overview of the types, sizes, and applications of NP-embedded MOF is provided in Table 2. Notably, when the metal precursor concentration exceeds the MOF’s intrinsic adsorption capacity, kinetic control is challenged by diffusion limitations. This can lead to heterogeneous nucleation at preferential sites that present a lower energy barrier, disrupting uniform growth. Subsequently, NP growth is anisotropically guided by the MOF’s pore–wall chemistry, wherein functional groups modulate the local environment to selectively stabilize specific NP facets. Ultimately, it is the synergy of nanoscale confinement and surface chemistry—rather than pore size alone—that finely controls the kinetics. This synergy restricts mass transport and Ostwald ripening, yielding ultrasmall, uniform NPs with unique atomic ordering, which is key to superior catalytic performance.

### 2.4. NP-Decorated MOF

Similarly to the NP-embedded MOF method, the NP-decorated MOF method is also based on pre-synthesized MOFs. Its core advantage lies in maximally retaining the crystal structure and porosity of MOFs. Unlike embedding within pores, this method loads plasmonic NPs onto the surface of MOFs or at the pore openings, which is more suitable for constructing open-type heterogeneous interfaces. According to the mechanism of action, it can be divided into two strategies: physical adsorption and chemical reduction.

#### 2.4.1. Physical Adsorption

This strategy aims to regulate the electrostatic matching between the surface charge properties of MOFs and plasmonic NPs. By means of non-covalent bonding such as electrostatic interactions, plasmonic NPs spontaneously adsorb onto the MOF surface or pore openings. This approach effectively solves the dispersion stability problem of plasmonic NPs without compromising the structural integrity of MOFs.

For example, after electrodepositing an HKUST–1 thin film on a copper foil substrate, Zhang et al. immersed it in a negatively charged silver colloid solution [74]. Uniform anchoring of Ag NPs on the HKUST–1 surface was achieved by utilizing electrostatic attraction between the positively charged metal nodes of HKUST–1 and the negatively charged Ag NPs. Comprehensive SEM characterization confirmed this structure: electrodeposited HKUST–1 exhibited regular octahedra with smooth surfaces, while the spherical Ag NPs were densely and uniformly distributed on the MOF without aggregation. The interfacial electronic interaction was further verified by XPS, which showed a 0.6 eV binding energy shift for Ag^0^ in the composite compared to pure Ag colloids, indicating charge transfer. This highly dispersed and orderly arrangement led to a stronger LSPR coupling effect, resulting in significantly enhanced SERS activity of the composite substrate.

To further improve the dispersion of NPs, Xu et al. synthesized 2D Zn–MOF nanosheets with a high specific surface area and combined them with positively charged gold nanostars (Au NSs, ζ–potential of +8.84 mV) through electrostatic self-assembly [75]. The ζ–potential of the final composite system was –4.72 mV. Characterization confirmed that Au NSs were uniformly distributed on the Zn–MOF surface in the form of single particles. This precise arrangement enabled the composite material to generate a higher efficiency of hot-electron injection under light illumination, and its photocatalytic bactericidal rate was 2.5 times higher than that of the physically mixed system. By synergistically regulating the surface charge density of MOFs and the type of NP modifiers, the random defects associated with traditional electrostatic adsorption can be overcome, enabling the precise construction of the NP–MOF interface.

#### 2.4.2. Chemical Reduction

The in situ philosophy extends to the surface decoration of MOFs through chemical reduction. Unlike physical adsorption, this method constructs local active sites on the MOF surface (e.g., by anchoring reducing agents or metal ions) to guide the directional, on-site reduction of metal precursors into NPs. The core of this method lies in utilizing specific coordination or interactions between the MOF, reducing agents, and metal ions to construct localized reaction sites.

It is common practice to pre-immobilize metal ions on the surface of the MOF and then reduce them to metal NPs using a reducing agent. For example, Srabani et al. used AgNO_3_ and Na_2_PdCl_4_ as precursors and employed NaBH_4_ to reduce and load Ag/Pd bimetallic NPs on the surface of UiO–66–NH_2_ [76]. The XRD pattern remained largely unchanged after NP introduction, indicating a preserved and stable MOF structure. Under visible light, the catalytic rates of the composite material for H_2_O_2_ and H_2_ were significantly higher than those of the pure MOF. On the one hand, the LSPR effect of Ag NPs enhanced the visible-light absorption ability of the photocatalyst; on the other hand, the bimetallic surface could effectively capture photogenerated electrons and inhibit the recombination of photogenerated electron–hole pairs, thereby improving the photocatalytic efficiency.

For the anchoring of reducing agents, tannic acid (TA) is an ideal choice. TA contains multiple phenolic hydroxyl groups that form stable coordination bonds with MOF metal nodes. Its inherent reducibility induces directional reduction of metal ions on the MOF surface while stabilizing the MOF structure [77,78]. Building on these properties, Jiang et al. mixed TA with MIL–101(Fe), where the phenolic hydroxyl groups of TA formed stable coordination bonds with Fe^3+^ (Figure 3C) [67]. Upon subsequent introduction of an AgNO_3_ solution, TA served a dual function: as an adsorption site for Ag^+^ and as a reducing agent, inducing the in situ generation of Ag NPs on the MOF surface (Figure 3D,E). The resulting Ag@TA/MIL–101 composite material can perform ultrasensitive detection of dopamine.

The NP-decorated MOF method achieves the controllable loading of plasmonic NPs on the open interface through two approaches: electrostatic matching via physical adsorption and coordination anchoring via chemical reduction, while preserving the intrinsic pore structure of MOFs. An overview of the types, sizes, and applications of NP-decorated MOF is provided in Table 3. However, this method has inherent limitations that can critically impact performance. First, the predominant distribution of NPs on the MOF’s external surface—a direct consequence of the synthesis strategy—inevitably poses a risk of pore aperture blockage. This physical obstruction can severely diminish the MOF’s intrinsic molecular sieve effect and active site accessibility, directly undermining its core functions of selective analyte enrichment and substrate-specific catalysis. Second, surface-located NPs create an additional diffusion barrier, impeding reactants from accessing the MOF’s internal active sites. This lengthens the diffusion pathway and increases mass transfer resistance, which is particularly detrimental in dynamic or cascade catalytic reactions where rapid substrate turnover is crucial. In such cases, these diffusion limitations can become the rate-determining step, effectively overshadowing the enhanced catalytic activity intended by the NP–MOF combination.

This section has elaborated on four primary synthetic methodologies for fabricating plasmonic NP-MOF hybrid materials. These strategies encompass encapsulation, iterative assembly, embedding, and surface decoration techniques. Each method entails a unique balance between synthetic complexity, precision of interfacial engineering, and the ultimate physicochemical characteristics of the resulting composites. A key advantage lies in the material versatility; the specific choice of plasmonic NPs can be tailored to target particular plasmonic responses, while the MOF matrix can be selected and engineered based on the dimensions and binding affinity of the guest molecules intended for interaction. Consequently, as will be illustrated in subsequent sections, the determination of the most suitable NP-MOF configuration is fundamentally guided by the performance demands of the intended practical application.

## 3. Applications

The combination of plasmonic NPs with MOFs facilitates precise analyte transport to high-field regions near plasmonic NPs through the efficient enrichment and selective adsorption of target molecules by MOFs. This spatial proximity amplifies the impact of local refractive index (RI) changes induced by molecular adsorption on the LSPR signals, thereby expanding the applications of traditional plasmonic sensing. Moreover, this hybrid structure harnesses a unique synergistic effect between NP and MOF components, enabling novel applications exclusive to the composite system. In this section, we will elaborate on how NP–MOF hybrids enhance existing plasmonic technologies and highlight new applications enabled by their synergy.

### 3.1. Chemical Sensing

**Figure 4:**
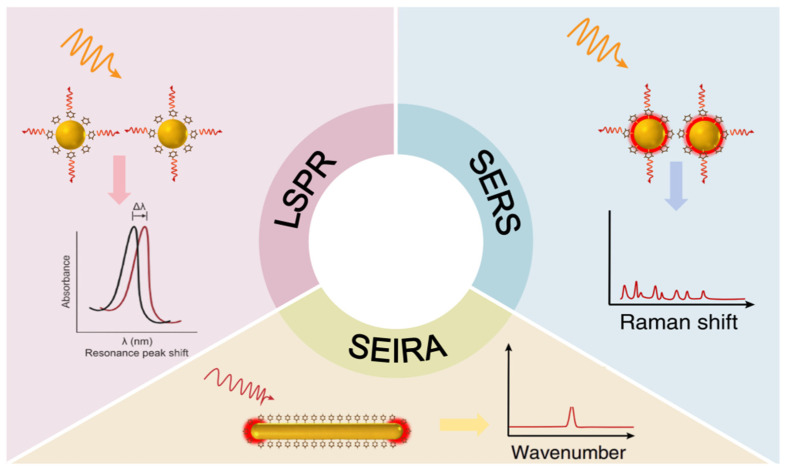
Principles of LSPR, SERS, and SEIRA.

The detection of trace compounds is vital for environmental protection and human health. Three prominent plasmonic sensing techniques—LSPR, SERS, and SEIRA—address the associated sensitivity challenges. Operating on distinct yet complementary principles, they constitute versatile sensing platforms, with their fundamental mechanisms illustrated in Figure 4.

LSPR sensing relies on analyte adsorption, which alters the local RI around NPs and induces a measurable shift in the plasmon resonance peak. Typically employing NPs with relatively large interparticle spacing, LSPR offers rapid response, operational simplicity, and label-free detection. However, its selectivity towards structurally similar molecules is often limited, making it ideal for applications such as real-time environmental monitoring and rapid water quality screening [79]. In contrast, SERS achieves substantial signal enhancement through intense EM fields localized at sharp nanotips and within ultra-narrow interparticle gaps. Its key strengths include an ultra-low detection limit, a high signal-to-noise ratio, and the provision of unique molecular fingerprint information. A notable drawback is the poor stability and inconsistent reproducibility often associated with tip-rich nanostructures [80,81]. Despite this, SERS remains a powerful tool for trace biomarker detection and food contaminant analysis. SEIRA, meanwhile, utilizes plasmonic nanorods whose resonance modes are tunable via aspect ratio to match specific molecular IR absorption bands. This technique provides high selectivity, enables non-destructive analysis, and yields valuable molecular structure information. Its main limitations are a lower enhancement factor compared to SERS and more stringent sample preparation requirements. SEIRA finds primary application in toxic gas detection and the structural identification of organic pollutants [82,83].

**Figure 5:**
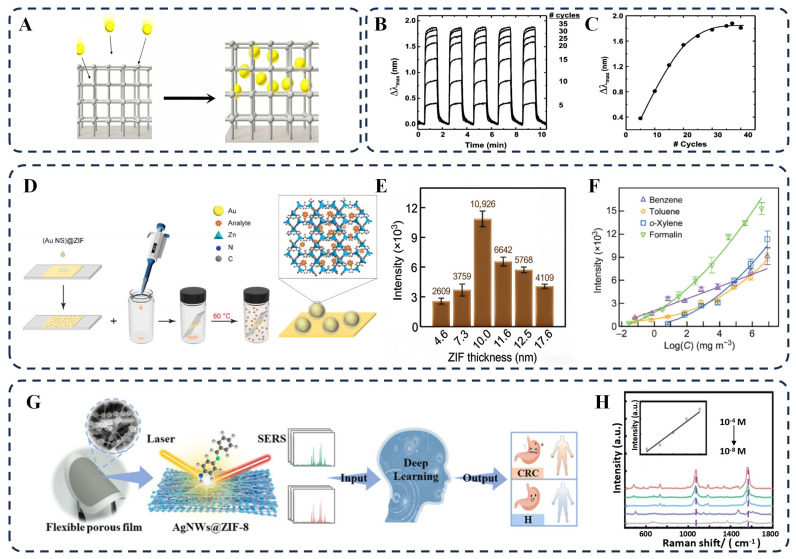
(**A**) Schematic of the molecular preconcentration effect by MOFs. (**B**) Dynamic response of Ag NPs@HKUST-1 sensors to pulsed CO_2_. (**C**) Enhancement of sensor response (Δλmax) with increasing HKUST-1 growth cycles. (**D**) Mechanism of VOC enrichment into plasmonic hotspots by a ZIF-8 shell. (**E**) Optimization of SERS enhancement by tuning ZIF-8 shell thickness. (**F**) Concentration-dependent SERS intensities for multiple VOC analytes. (**G**) Ag NWs@ZIF-8 SERS sensor for breath-based cancer screening via deep learning. (**H**) Wide-range calibration curve for toxic VOCs with an ultralow detection limit. Reprinted and adapted with permission from (**B**,**C**) reference [85], (**D**–**F**) reference [15], and (**G**,**H**) reference [26]. Copyright 2010 and 2024 American Chemical Society.

A fundamental challenge in trace detection stems from the ultralow concentrations of target analytes, their weak inherent affinity for plasmonic surfaces, and small optical cross–sections, collectively resulting in feeble signals [84]. Critically, all the above techniques depend on the close proximity of analytes to the nanoparticle surface for effective signal generation. To overcome this limitation, the integration of MOFs presents a powerful strategy. The porous MOF matrix, characterized by high surface area and tunable pores, efficiently adsorbs and preconcentrates target molecules, delivering them to the high-field regions adjacent to plasmonic NPs (Figure 5A) [11]. This targeted enrichment within EM hot spots dramatically amplifies the detection signal. By synergistically combining the superior adsorption capacity of MOFs with the tunable optical properties of plasmonic nanostructures, NP–MOF composite platforms offer a highly sensitive and versatile solution for trace compound detection.

#### 3.1.1. LSPR Sensing

LSPR-based sensing is widely used in molecular detection due to its sensitivity to RI changes. When the analyte comes into contact with plasmonic NPs, the resultant RI change leads to shifts in LSPR peak wavelength, enabling quantitative analysis of the target [86]. However, this technology faces significant challenges in the field of gas sensing. On one hand, small-molecule gases typically induce only weak spectral shifts of approximately 0.02 nm, whereas macromolecular substances such as proteins can produce shifts as large as 8.5 nm [87]. On the other hand, target gas concentrations in practical applications are typically in the parts-per-billion (ppb) range, demanding low limits of detection (LOD) [88,89].

To address this, Kreno et al. fabricated arrays of Ag NPs via nanosphere lithography and then deposited HKUST–1 films using layer-by-layer self-assembly to control thickness [85]. HKUST–1 selectively concentrated CO_2_ within its pores, increasing the local RI and inducing a redshift of the LSPR peak (Figure 5B). The LSPR peak shift increased with the number of HKUST–1 growth cycles, but the enhancement tapered off as the film approached the effective sensing volume (Figure 5C). Specifically, Δλmax rose rapidly at low growth cycles—when new MOF growth was closest to the Ag nanoparticle surface—and reached a maximum of 1.88 nm at 37 cycles, representing a 14-fold enhancement in the LSPR response compared to bare Ag NPs. This trend arises from the exponential decay of the plasmon-induced EM field from the nanoparticle surface; molecules near the surface dominate the signal response, so additional adsorption capacity from thicker films beyond the effective sensing volume does not significantly improve sensitivity.

Building on the concept of MOF-enhanced sensing, Guo et al. constructed a more complex composite interface to harness the ultrahigh surface area and selective adsorption of MOFs more effectively. They developed a multi-level enhanced LSPR sensor based on Au nano-urchins for highly sensitive gas detection [90]. After depositing Au nano-urchins on a substrate, ZIF–8 was grown on the surface, and the resulting composite was integrated with PEDOT:PSS. Synergy between ZIF–8 and PEDOT:PSS enabled sensitive ammonia detection. ZIF–8 functions as a “molecular sponge” to enrich gas molecules on nano-urchins, thereby enhancing the sensitivity of the sensors by 5 to 7 times compared with unmodified sensors.

For the detection of macromolecules, the MOF–LSPR composite system also demonstrates unique advantages. The Au NPs/Cu–TCPP/Au sensor developed by Gao et al. achieved ultrasensitive dopamine detection by coupling SPR and LSPR [91]. When the probe DNA captures dopamine, the RI change can be amplified by the local field of Au NPs, and the response signal is further enhanced through the 2D MOF layer. This sensor outperformed non-amplified structures in sensitivity and figure of merit (FOM). Luan et al. combined Molecularly Imprinted Polymers (MIP) with MOFs to achieve highly sensitive detection of macromolecular proteins [92]. By constructing Human Serum Albumin (HSA) imprinted cavities on Au NRs and subsequently growing ZIF–8, they enhanced the RI gradients after target capture. MOF modification doubled sensitivity and reduced the LOD to 33% of the original value. This strategy was also successfully extended to lysozyme and hemoglobin, broadening LSPR applications for non-antibody-dependent detection.

Although introducing MOFs significantly enhances LSPR sensor performance, the technology faces a fundamental limitation: its signal lacks inherent molecular specificity. Since LSPR shifts only measure bulk RI changes, the technique suffers from pronounced cross-sensitivity; it cannot distinguish between different analytes or interferents that induce similar RI changes in the local environment. This fundamental constraint limits its selectivity in complex mixtures and hinders its application in real-world samples where multiple components coexist. Addressing this challenge requires strategies that move beyond the intrinsic physical nature of LSPR. Future research should therefore focus on integrating LSPR with orthogonal techniques. For example, coupling LSPR with surface-enhanced vibrational spectroscopy (SERS/SEIRA) could merge high sensitivity with molecular fingerprinting, enabling precise discrimination and effectively deconvoluting signals from mixed analytes.

#### 3.1.2. SERS Sensing

Surface-enhanced Raman scattering (SERS) technology overcomes the inherent lack of molecular specificity in LSPR sensing by providing vibrational fingerprint information coupled with exceptional signal amplification, which originates from both EM and chemical (CM) enhancement mechanisms [93,94]. The integration of MOFs into these systems profoundly augments this dual-path enhancement through multifaceted mechanisms that extend beyond mere preconcentration. Specifically, the MOF shell modulates the local dielectric environment around the plasmonic nanoparticle, perturbing its plasmonic resonance. This perturbation can reconfigure and concentrate the EM field within the MOF’s porous architecture, thereby positioning analyte molecules more effectively within intensified EM “hot spots” [50,95]. Beyond this physical effect, the MOF’s tunable electronic structure allows it to function as an active charge-transfer mediator. By aligning its energy levels between the nanoparticle and the adsorbed analyte, the MOF facilitates the efficient transfer of photoinduced hot carriers, providing a direct and synergistic boost to the chemical enhancement mechanism [96]. Thus, the MOF porosity directly engineers the local photonic density of states for EM enhancement, while its tailored electronic structure creates dedicated pathways for CM, moving beyond its traditional roles of mere stabilization and enrichment.

The multifunctional role of MOFs in SERS platforms extends beyond signal amplification to encompass critical performance attributes such as enhanced stability and selective enrichment. For instance, Ag NWs coated with ZIF–8 maintain SERS activity in high–temperature, high-humidity, and enzyme-rich environments [97]. This protective effect arises from the physical barrier MOFs provide against reactive oxygen species and their coordination interaction with the metal surface. Regarding selective enrichment, MOFs leverage their high specific surface area, π–π interactions, and hydrophilicity/hydrophobicity to specifically adsorb target molecules, thereby enhancing the specificity and sensitivity of SERS [98,99,100]. A representative example is the shell of MOF–5 coated on Au NPs, which selectively adsorbs CO_2_, enabling its detection in gas mixtures and producing a significantly enhanced Raman signal at 1395 cm^−1^ [101]. The signal intensity depends on the MOF–5 shell thickness, peaking at approximately 3.2 nm, while an overly thick coating can diminish SERS performance by shielding the light absorption of the plasmonic NPs.

Function-oriented composite material design demonstrates significant application potential. For complex sample systems, a Fe_3_O_4_–Au@MIL–100(Fe) composite integrates multiple functions: the magnetic Fe_3_O_4_ core facilitates rapid separation and uniform deposition, Au NPs provide EM enhancement, and MIL–100(Fe) contributes chemical enhancement and analyte adsorption [102]. This synergistic system achieved detection limits of 4.4 nM for Malachite Green and 15 nM for Thiram, demonstrating a linear response in real water samples and good reusability.

In constructing plasmonic hotspots and molecular enrichment, Liu et al. developed a “nanoparticle-on-mirror” substrate based on a Au nanocrystal@ZIF–8 core–shell structure [15]. By growing a ZIF–8 shell on Au nanocrystals regulated by surfactants and drop–casting them onto a gold film, strongly coupled hotspots were formed. The ZIF–8 shell acts simultaneously as a nanogap tuner and a molecular trap, enriching VOCs into the hotspot region (Figure 5D). By optimizing preparation parameters, optimal SERS enhancement was achieved at a ZIF–8 thickness of 10 nm (Figure 5E), enabling the quantitative detection of toxic VOCs across 8 orders of magnitude with a detection limit as low as 10^−2^ mg/m^3^ (Figure 5F).

For flexible sensing and disease screening, Li et al. grew a hierarchical porous ZIF–8 coating resembling a “candied haws” string on Ag NWs to create a flexible SERS film [26]. This porous structure enhances gas adsorption capacity and generates unique and stable SERS spectra (Figure 5G). For example, when the composite binds with dithio–p–benzoquinone (DP), the Raman peak intensity at 1070 cm^−1^ increases significantly, showing a linear relationship at low concentrations (Figure 5H). This system can also detect other molecules through changes in Raman peak intensity at specific wavelengths. Combined with an artificial neural network algorithm, it achieved a recognition accuracy of 93.7% for colorectal cancer markers in simulated exhaled breath, offering a new approach for early disease screening.

Although the introduction of MOFs has significantly improved SERS performance, several bottlenecks require further attention. First, the limited selectivity for isomers stems not only from comparable molecular sizes but, more fundamentally, from the non-specific nature of common host–guest interactions (e.g., π–π stacking) within MOF pores. Introducing specific functional groups via post-synthetic modification is thus crucial to enhance chemical recognition. Second, the inherent Raman signals of MOFs can create a non-negligible spectral background. This highlights a trade-off: the MOF, while functional, can itself become an interference source, necessitating systematic evaluation of its background during substrate design. Finally, the application-dependent chemical stability of many MOFs presents a material-level contradiction: the very porosity and active sites that enable superior enrichment and chemical enhancement also expose the framework to degradation, demanding innovative stabilization strategies for real-world deployment.

#### 3.1.3. SEIRA Sensing

SEIRA-based sensors enable the identification of various gaseous analytes (e.g., CO_2_, CH_4_) and provide sensitive gas-phase analysis capabilities [103,104]. MOFs act as molecular concentrators, enriching target molecules within the plasmonic near-field through pore-size screening and chemical adsorption, while plasmonic nanostructures amplify molecular IR signals via localized EM field enhancement [105]. To achieve optimal sensing performance, two key aspects require optimization: first, matching the MOF pore size to the target molecules and adjusting the MOF layer thickness to avoid optical transmission loss; second, designing plasmonic metamaterials with multiple resonance characteristics to precisely couple their operational bands with the vibrational signature bands of the target molecules [106].

**Figure 6:**
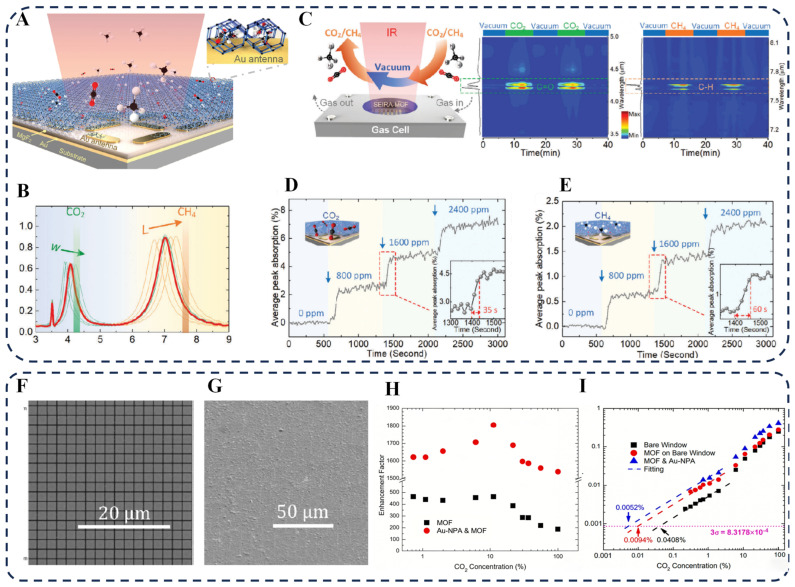
(**A**) Schematic representation of the MOF–SEIRA platform consisting of porous ZIF–8 and a metamaterial perfect absorber. (**B**) Simulated absorption spectrum of the MOF–SEIRA platform. (**C**) Dynamic characteristics of the proposed MOF–SEIRA platform for simultaneous sensing of CO_2_ and CH_4_. Dynamic behavior of MOF–SEIRA platform as (**D**) CO_2_ and (**E**) CH_4_ concentrations increase. SEM image of Au–NPA (**F**) before and (**G**) after coating the MOF thin film with the corresponding concentration. (**H**) Calculated enhancement factor as a function of CO_2_ concentration. (**I**) Detection limit based on noise analysis. Reprinted and adapted with permission from (**A**–**E**) reference [107] and (**F**–**I**) reference [108]. Copyright 2020 Wiley-VCH Verlag. Copyright 2017 American Chemical Society.

Through ingenious structural design, the performance of SEIRA sensors can be significantly enhanced. For example, Zhou et al. proposed a novel strategy by constructing a Fabry–Pérot cavity using symmetrical cross–shaped gold nanoantennas and integrating it with ZIF–8, enabling simultaneous detection of two gases (Figure 6A) [107]. The 3.4 Å micropores of ZIF–8 preferentially capture CO_2_ molecules, while its 11.6 Å cavities enrich CH_4_ molecules, thereby mitigating competitive co-adsorption between them (Figure 6B,C). The resonance bands of the Fabry–Pérot cavity were designed to align with the characteristic absorption bands of CO_2_ (2350 cm^−1^) and CH_4_ (1305.9 cm^−1^), respectively. As the concentrations of CO_2_ and CH_4_ increased from 0 to 2400 ppm, the sensor’s absorption signal showed a marked rise at each concentration step before stabilizing, with response times of 35 s for CO_2_ and 60 s for CH_4_ (Figure 6D,E). This design provides a new paradigm for real-time multi-component gas monitoring.

Another effective strategy involves achieving full-range field enhancement by optimizing the substrate and composite structure. Chong et al. integrated gold nanopatch antennas (Au NPAs) with a ZIF–8 thin film on a Si_3_N_4_ nanomembrane substrate, achieving light field enhancement with a higher quality factor [108]. SEM images show the morphology of the Au NPAs before and after MOF coating, confirming that the MOF layer fully covers the Au NPAs to form a stable hybrid structure (Figure 6F,G). Modification with Au NPAs and MOF significantly improved the sensor’s CO_2_ response sensitivity. As shown in Figure 6H, combining the gas concentration effect of MOF and plasmonic field enhancement resulted in an overall enhancement factor exceeding 1800. Figure 6I demonstrates a detection limit as low as 52 ppm. Due to the surface properties of ZIF–8, water molecules are mainly adsorbed on its outer surface, while CO_2_ molecules can diffuse into the inner pores, thereby endowing the sensor with good stability in high-humidity environments. This full-range field enhancement strategy paves a new path for developing miniaturized on-chip SEIRA sensors.

Although the introduction of MOFs has significantly improved SEIRA sensor performance, several key bottlenecks require critical analysis. First, the sharp decline in IR transmittance of dense MOF films in the long-wavelength region is not merely an optical issue but fundamentally limits the detection of larger molecules (e.g., VOCs) whose characteristic vibrations fall within this “fingerprint” region. This attenuation stems from the intrinsic vibrational absorption and scattering losses of the MOF framework itself. Therefore, mitigation strategies must focus on material architecture, such as synthesizing hollow or ultrathin MOFs to reduce optical path length, or introducing graphene interlayers to enhance mid-infrared light coupling. Second, the unresolved issue of cross-sensitivity in multi-gas detection highlights a deeper material design challenge: while MOFs enrich gases, their conventional pore structures often lack the precise chemical discrimination needed for complex mixtures. Overcoming this limitation may necessitate developing hierarchically porous MOFs combined with machine learning (ML) algorithms to achieve spectral deconvolution [109]. Third, regarding long-term stability, MOF structural collapse during cyclic adsorption–desorption is a critical failure mode that undermines sensor reliability. This instability often originates from the labile coordination bonds at metal nodes under repetitive stress. A promising reinforcement strategy involves introducing coordination additives (e.g., modulating ligands or metal ions) to strengthen the framework and enhance cyclic stability, ensuring practical deployment [110].

### 3.2. Cancer Therapy

Globally, infectious and malignant diseases, including various cancers, represent a significant challenge to human health. The treatment of cancer remains particularly difficult, even with the widespread use of numerous anticancer modalities such as surgical intervention, chemotherapy, radiation therapy, thermal ablation, and immunotherapy. Approaches relying on a single therapeutic method often demonstrate not only restricted treatment efficacy but also considerable adverse effects, which can encompass harm to healthy tissues and an elevated likelihood of disease recurrence. Consequently, the development of multi-modality therapeutic platforms that efficiently integrate diverse treatment strategies has emerged as a predominant approach for achieving synergistic effects in cancer management.

#### 3.2.1. Chemo–Photothermal Therapy

MOFs serve as effective drug carriers due to their ability to encapsulate chemotherapeutic agents within porous frameworks and release them in response to specific stimuli (e.g., pH, temperature). Their inherent biodegradability and biocompatibility further support biomedical applications [111]. For instance, ZIF–8 remains stable under physiological conditions but degrades in acidic tumor microenvironments, enabling targeted drug release. Moreover, plasmonic NPs can potentiate this process by converting light into heat, thereby enhancing therapeutic outcomes.

**Figure 7:**
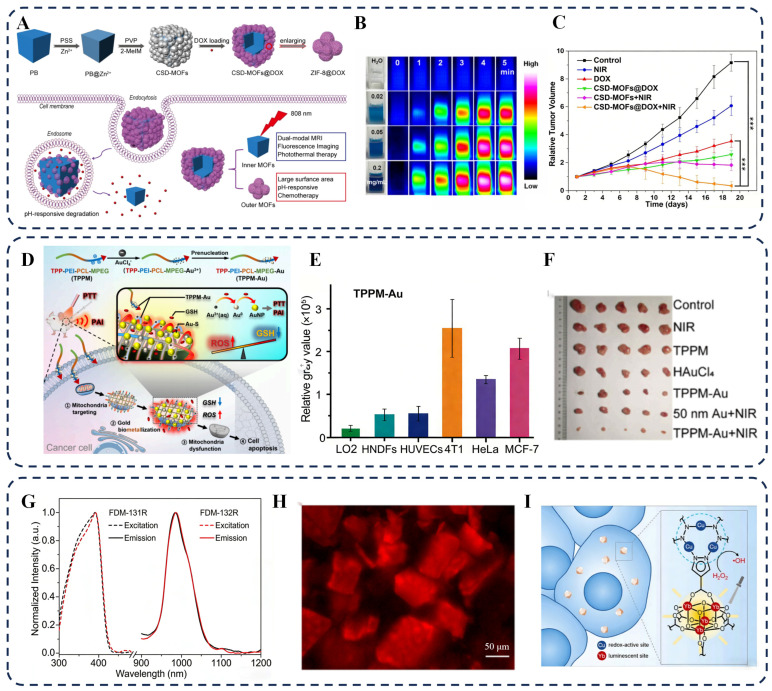
(**A**) Schematic of the NP-MOF hybrid system for synergistic tumor therapy. (**B**) Photograph of excised tumors from mice. (**C**) Relative tumor growth curves under different treatments (*** indicates a statistically significant difference with *p* < 0.001). (**D**) Mechanism of the mitochondria-targeted TPPM-Au therapeutic system. (**E**) Cancer cell-specific enhancement of biometallization efficiency. (**F**) Tumor photographs highlighting the optimal treatment group. (**G**) Photothermal heating curves under NIR laser irradiation. (**H**) Cyclic photothermal stability test. (**I**) Photoacoustic signal intensity under laser excitation. Reprinted and adapted with permission from (**A**–**C**) reference [27], (**D**–**F**) reference [28], and (**G**–**I**) reference [113]. Copyright 2017 Ivyspring International Publisher. Copyright 2023, 2024 American Chemical Society.

Demonstrating this synergy, Wang et al. developed a core–shell Prussian blue@ZIF–8 (CSD–MOFs) nanotheranostic agent [27]. The clear core–shell structure of the synthesized material was confirmed by characterization shown in Figure 7A. This system utilizes the large surface area and pH responsiveness of the ZIF–8 shell to achieve ultrahigh loading of doxorubicin (DOX) and pH/NIR laser dual-stimuli responsive release. Under 808 nm NIR laser irradiation, the CSD–MOFs exhibited efficient photothermal conversion, as visually demonstrated by the temperature increase captured via IR thermal imaging in Figure 7B. The generated heat not only directly kills cancer cells but also accelerates the degradation of the ZIF–8 shell and drug release in the acidic tumor microenvironment, thereby achieving synergistic chemo–photothermal therapy. The superior therapeutic outcome of this combined approach was conclusively proven in vivo, where the group treated with “CSD–MOFs@DOX + NIR” showed the most effective tumor growth suppression, as evidenced by the tumor growth curves in Figure 7C.

The limited stability of the ZIF–8 matrix in biological media can be improved by coating with an amphiphilic polymer shell to obtain highly stable thermoresponsive plasmonic nanocarriers [112]. This structure enhances drug loading capacity and targeted release efficiency, while further improving therapeutic efficacy via NIR-light-triggered photothermal effects. It enables efficient drug release and local hyperthermia at tumor sites, significantly improving treatment outcomes and reducing damage to normal tissues, thereby offering new possibilities for personalized cancer therapy.

#### 3.2.2. Photothermal Therapy

Photothermal therapy (PTT) is an emerging treatment approach that has gained significant attention in cancer therapy. It utilizes light-induced hyperthermia to target local malignant tissues and eliminate abnormal cells [114]. Moreover, plasmonic photothermal therapy (PPTT), which employs plasmonic nanocomposites, exhibits superior anticancer efficacy compared with traditional laser treatments. Luo et al. recently reported an innovative mitochondria-targeted strategy using a polymer–gold complex (TPPM–Au) to achieve in situ gold biometallization for enhanced photothermal cancer therapy [28]. The design and working principle of this system, wherein the complex delivers gold ions to mitochondria for the formation of photothermal Au NPs, is illustrated in Figure 7D. A key advantage of this strategy is its high selectivity for cancer cells. Quantitative analysis in Figure 7E demonstrates that the biometallization efficiency in various cancer cell lines (4T1, HeLa, MCF–7) was 3 to 6 times higher than in normal cells, due to the higher redox environment in cancerous mitochondria. This in situ GNP formation consumes mitochondrial GSH, amplifies oxidative stress, and concurrently generates NPs with a high photothermal conversion efficiency of 48.6%. The superior in vivo therapeutic outcome of combining this targeted biometallization with PTT is conclusively demonstrated in Figure 7F. Photographs of excised tumors show that the group treated with “TPPM–Au + NIR” exhibited the smallest tumor size among all groups, providing direct visual evidence of the platform’s potent and selective tumor ablation capability.

#### 3.2.3. Molecular Imaging

Traditional imaging methods such as photoacoustic imaging (PAI) and computed tomography (CT) suffer from poor spatial resolution and limited accuracy in tumor diagnosis [115,116]. To overcome these challenges and achieve precise theranostics, a significant research direction focuses on engineering unified material platforms that intrinsically combine imaging and therapeutic functions, as opposed to relying on heterogeneous nanocomposites, which may have inconsistent behavior in vivo.

TA’s prime example of this design philosophy is presented by Yang et al., who developed multinary MOFs with atomically precise integration of near-infrared (NIR) imaging and chemodynamic therapy (CDT) capabilities [113]. They constructed crystalline MOFs (FDM–131 and FDM–132) incorporating both luminescent Yb-based clusters and redox-active Cu-based nodes within a single, robust framework. The Yb units confer excellent imaging properties, characterized by a distinct NIR emission peak at 986 nm upon excitation (Figure 7G). This intrinsic luminescence allows for direct visualization, as demonstrated by the clear NIR epifluorescence signal from individual MOF crystals shown in Figure 7H. The core innovation lies in the simultaneous and spatially consistent operation of imaging and therapy. As illustrated in the schematic Figure 6I, the Cu(I)/Cu(II) sites catalytically convert tumor-overexpressed H_2_O_2_ into cytotoxic hydroxyl radicals for CDT, while the Yb(III) sites provide the NIR emission for imaging—all within the same, stable particle. This synergy ensures that the therapeutic action and the imaging signal originate from an identical location, enabling high-fidelity monitoring of the treatment agent. After cellular uptake, these MOF NPs not only show strong intracellular NIR signals but also efficiently elevate reactive oxygen species levels, leading to significant cancer cell apoptosis.

Therefore, compared to conventional imaging or composite agents, such rationally designed, multifunctional MOFs represent an advanced strategy. They provide not only enhanced imaging contrast but also guarantee a unified theranostic mechanism with high spatiotemporal consistency, paving the way for more precise and reliable tumor diagnosis and therapy. Despite the promising therapeutic outcomes demonstrated by these NP–MOF hybrid platforms, a critical evaluation of their long-term biocompatibility and in vivo fate is essential for clinical translation. The very properties that make them effective—such as the high surface area for drug loading, the metallic components for photothermal conversion, and the responsive degradation for drug release—also raise important biosafety considerations. First, the degradation kinetics and clearance pathways of MOFs (e.g., ZIF–8 in acidic lysosomes) must be meticulously tuned to ensure that the released metal ions and organic linkers are non-toxic and efficiently excreted [117]. Second, while noble metal NPs like Au are generally considered biocompatible, their long–term accumulation and potential immune response require thorough investigation, especially for systemic administration [118]. Future material design should proactively address these challenges by integrating inherently safer components (e.g., Fe–, Zn–, or Zr–based MOFs), engineering surface coatings to evade immune recognition and enhance renal clearance, and establishing rigorous structure–biocompatibility–activity relationships [119,120]. Only by advancing such a holistic design paradigm can these sophisticated theranostic platforms transition from compelling laboratory demonstrations to viable clinical solutions.

### 3.3. Catalysis and Adsorption

This section focuses on the applications of NP–MOF composites in three major fields: electrocatalysis, photocatalysis, and adsorption–separation. In electrocatalysis, structural design optimizes active sites and reaction selectivity, demonstrating excellent performance in scenarios such as water disinfection and nitrogen fixation. In photocatalysis, plasmonic effects and heterojunction construction synergistically enhance light absorption and charge separation efficiency. For adsorption–separation, materials achieve efficient pollutant adsorption, multi-phase separation, and recycling through the synergistic effects between MOFs and NPs. Additionally, this section highlights key challenges in current research, including mechanism elucidation and large-scale preparation.

#### 3.3.1. Electrocatalysis

Electrocatalysis, as a mild and efficient technology, holds significant application value in key areas such as drinking water disinfection and green ammonia synthesis, offering new solutions to global water security and energy transition challenges. However, traditional electrocatalysts are often limited by insufficient active sites, poor selectivity, and interference from competitive reactions. MOFs, with their controllable porous structures, high specific surface areas, and molecular enrichment effects, can form composite systems with nano-active components to optimize electrocatalytic performance through synergistic effects, providing an effective strategy to overcome these traditional bottlenecks.

**Figure 8:**
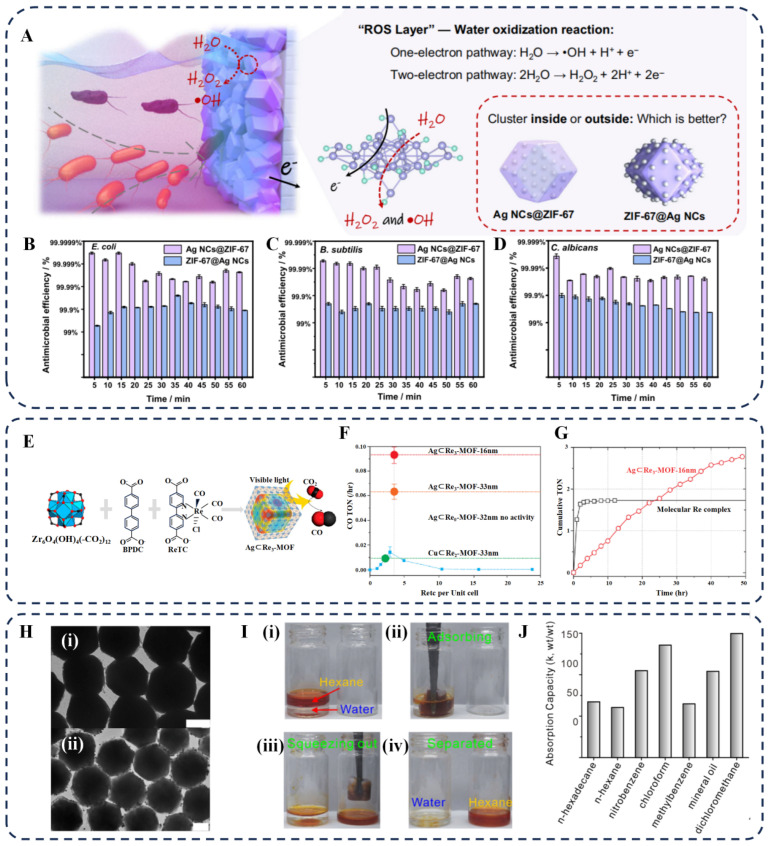
(**A**) Working mechanism of the CEWD system featuring a cyclic “capture–kill–desorb” process. (**B**–**D**) Broad-spectrum microbial killing performance of CEWD, with rates >99.9% against *E. coli*, *B. subtilis*, and *C. albicans*. (**E**) Preparation and catalytic principle of the Ag⊂Re_3_–MOF structure. (**F**) Photocatalytic CO_2_-to-CO conversion activity of Ren-MOFs (blue line), Ag⊂Re_0_-MOF, Cu⊂Re_2_-MOF, and Ag⊂Re_3_-MOFs with MOF thickness of 16 and 33 nm. (**G**) Cycling stability comparison between Ag⊂Re_3_–MOF and a molecular catalyst. (**H**) TEM images of (i) ZIF-8 (scale bar: 1 μm) and (ii) ZIF-8@GNPs-PFDT (scale bar: 1 μm). (**I**) Photographs of the oil/water separation process using the superhydrophobic composite-coated foam: (i) The hexane/water mixture with hexane dyed by Sudan I and water in a beaker; (ii) The ZIF-8@GNPs-PFDT foam is immersed into the mixture, and the dyed hexane starts to be adsorbed by the foam; (iii) The foam fully adsorbs the hexane and is taken out from the beaker, leaving the colorless aqueous phase in the beaker; (iv) The adsorbed hexane is recovered by squeezing the foam. (**J**) Adsorption capacities of the foam for various organic solvents and oils. Reprinted and adapted with permission from (**A**–**D**) reference [121], (**E**–**G**) reference [29], and (**H**–**J**) reference [30]. Copyright 2023 and 2024 American Chemical Society. Copyright 2017 Springer Nature.

MOF composites incorporating atomically precise nanoclusters have demonstrated remarkable advantages in electrochemical water treatment. For instance, a capacitive-charging induced electrochemical water disinfection (CEWD) system was developed using a composite electrode material of Ag_28_ nanoclusters embedded in ZIF–67 (Ag NPs@ZIF–67) [121]. The synergy in this system is threefold (Figure 8A): the porous structure of ZIF–67 provides abundant electrosorption sites while confining the Ag nanoclusters to prevent aggregation; the Ag_28_ nanoclusters act as electrocatalytic sites for the in situ generation of residue-free reactive oxygen species (ROS) while possessing intrinsic antimicrobial activity; combined with the electrostatic adsorption of negatively charged microbes, it enables a cyclic “capture–kill–desorb” process. This CEWD system exhibits excellent broad-spectrum microbial inactivation efficiency, achieving killing rates exceeding 99.99% for *Escherichia coli*, 99.90% for *Bacillus subtilis*, and 99.90% for *Candida albicans* (Figure 8B–D). Additionally, it features good stability over multiple cycles, low energy consumption, and no disinfection byproducts, making it suitable for various real water samples.

Through ingenious core–shell structure design, MOF-based nanocomposite systems have also achieved breakthroughs in energy-related reactions like the electrocatalytic nitrogen reduction reaction (NRR). An example is a catalyst with nanoporous gold (NPG) cores embedded in a ZIF–8 shell [122]. Here, the synergy addresses key NRR challenges: the porous NPG core provides a high density of active sites, while the hydrophobicity of the ZIF–8 shell significantly suppresses the competing hydrogen evolution reaction (HER), and its porosity enriches N_2_ molecules near the active sites, enhancing NRR kinetics. This composite catalyst demonstrates outstanding NRR performance under ambient conditions, including high ammonia yield and Faradaic efficiency, along with good stability.

Despite the great potential of MOF-based nanocomposites in electrocatalysis, current research still faces key challenges. Firstly, the adsorption/activation pathways of target molecules and the electron transfer mechanisms at the interface between MOF matrices and nano-active components require deeper clarification. Secondly, developing strategies to enhance the electrical conductivity of MOF-based structures while maintaining their porosity and functionality is crucial for adapting to more complex electrocatalytic scenarios. Additionally, achieving the large-scale preparation and engineering application of these catalysts is essential for their transition from the laboratory to practical use.

#### 3.3.2. Photocatalysis

Synergistic interactions between MOFs and plasmonic NPs enhance photocatalysis by merging complementary functions: plasmonic NPs harvest light via LSPR and generate hot electrons, while MOFs enrich reactants and stabilize active sites [123]. With high specific surface areas and designable pores, MOFs offer unique advantages in pollutant degradation (e.g., UV-driven tetracycline degradation by MOF(Fe)/FeS_x_), dye treatment (e.g., visible-light rhodamine B degradation by Ce–MOF/h–CeO_2_), and gas purification (e.g., photocatalytic methyl mercaptan decomposition by HKU@MIL) [124,125,126]. However, single-component materials suffer from narrow photoresponse ranges, high carrier recombination rates, and insufficient stability. NP–MOF composite systems achieve performance breakthroughs via functional complementarity: plasmonic NPs enhance light absorption and inject hot electrons through LSPR, while MOFs enrich reactants via selective adsorption and stabilize active sites, creating efficient catalytic microenvironments [127]. Additionally, doping metal ions into plasmonic NP–MOF composites has been shown to strengthen synergies and further boost photocatalytic efficiency [128].

To enhance photocatalytic performance, researchers have developed several key strategies.

Strategy 1: Utilizing spatial confinement to stabilize active species. Choi et al. covalently incorporated the photocatalyst Re(CO)(BPYDC)Cl (ReTC) into a UiO–67–based MOF (Re–MOF). By precisely controlling its pore density, they effectively suppressed the deactivation of the Re catalyst via dimerization, significantly improving the activity and stability of photocatalytic CO_2_ reduction (Figure 8E) [29]. Notably, the Ag⊂Re_3_–MOF structure (with a 16 nm–thick Re_3_–MOF coating) exhibited catalytic activity seven times higher than that of bulk Re_3_–MOF and maintained stable performance, whereas molecular H2ReTC rapidly deactivated due to dimerization, highlighting the advantage of this strategy for CO_2_ conversion (Figure 8F,G).

Strategy 2: Constructing heterojunctions to improve charge separation and transport. To address the poor intrinsic conductivity of MOFs, Liu et al. constructed a MOF–74/MIL–53 heterojunction via in situ growth. The intimate interfacial contact optimized the electronic structures of the metal centers and enabled fast charge transfer [129]. Under light irradiation, the Au–MOF–74/MIL–53 electrode exhibited an overpotential of only 218 mV at 50 mA cm^−2^, significantly lower than under dark conditions, along with a reduced Tafel slope, indicating improved reaction kinetics. This was primarily attributed to hot holes generated by Au NPs oxidizing Ni^2+^/Co^2+^ to the more OER–active Ni^3+^/Co^3+^.

Although NP–MOF hybrid structures have made appreciable advances in photocatalysis, several key issues require further investigation. For instance, the specific impact of electron transfer between NPs and MOFs on reactions, how to balance the roles of hot electrons and photogenerated carriers, and the mechanisms of energy transfer remain unclear. In-depth research into the synergistic effects between MNCs and MOFs and elucidation of the reaction mechanisms, while challenging, are essential for advancing their practical applications.

#### 3.3.3. Adsorption

Adsorption is a central functionality of NP–MOF composites, underpinning key applications in energy and environmental technologies such as gas storage and liquid-phase purification. Traditional adsorbents are often limited by poor selectivity and weak host–guest interactions. NP–MOF composites overcome these bottlenecks through synergistic effects: the high surface area and tunable pores of MOFs provide an ideal scaffold for molecular adsorption and sieving, while functional NPs actively regulate the pore environment, introduce catalytic sites, or modify surface properties, thereby enhancing selectivity, capacity, and performance in complex systems.

For gas storage, particularly hydrogen, NP–MOF composites achieve enhanced adsorption capacity via spillover effects. A notable example is the Pd@HKUST–1–DS composite [130]. Synergy arises from the spatial confinement of the HKUST–1 pores, which prevents Pd nanoparticle aggregation, and the hydrogen spillover effect catalyzed by the dispersed Pd NPs. In this process, H_2_ molecules dissociate on the Pd surface, and hydrogen atoms migrate and chemisorb onto the MOF framework, significantly increasing overall uptake. This material achieves a hydrogen adsorption capacity of 3.68 wt% at 77 K and 0.2 MPa. More importantly, it retains 1.63 wt% at 298 K/18 MPa, approximately 2.3 times and 1.4 times higher than pristine HKUST–1 and a comparator prepared by conventional impregnation, respectively.

In liquid-phase treatment, NP–MOF composites enable targeted pollutant removal through selective adsorption. A typical example is the Pd@MIL–100(Fe) composite designed for the uptake of pharmaceuticals and personal care products (PPCPs) [131]. The synergy is twofold: the ordered porous structure of MIL–100(Fe) provides abundant adsorption sites and confines Pd NPs against aggregation, while the Pd NPs optimize surface adsorption properties, enhancing selectivity and capacity for PPCPs in aqueous solutions.

Beyond pollutant capture, NP–MOF composites also address multiphase separation challenges by exploiting differential adsorption. The superhydrophobic ZIF–8@GNPs–PFDT composite demonstrates this in oil–water separation [30]. Its performance stems from two features (Figure 8H): the well–defined porous structure of ZIF–8 enables efficient adsorption and enrichment of hydrophobic substances with rapid mass transfer, while PFDT modification confers superhydrophobicity. This allows the composite–coated foam to effectively separate oil from water, as shown for a hexane/water mixture (Figure 8I). The PFDT layer selectively promotes adsorption of small hydrophobic molecules while blocking hydrophilic interferents. The composite exhibits a high oil adsorption capacity, stable performance over 10 cycles, and can be loaded onto foams or hydrogels for practical use (Figure 8J).

Despite these advances, several challenges remain. First, the fundamental mechanisms by which NPs modulate the MOF pore environment and synergistically enhance adsorption performance require deeper experimental and theoretical investigation [132]. Second, in practical applications, balancing high adsorption capacity, selectivity, and cycling stability while preserving MOF integrity is crucial [133]. Finally, scalable, low-cost synthesis and engineering of NP–MOF composites are essential for industrial adoption.

## 4. Outlook and Perspectives

Plasmonic NP–MOF hybrid systems represent a transformative platform in nanoscience, leveraging the synergistic integration of LSPR properties and MOF porosity to advance applications in catalysis, sensing, and biomedicine. However, transitioning these hybrids from fundamental research to widespread industrial deployment faces persistent, interconnected challenges. Chief among these are the scalable synthesis of stable architectures, the high cost of noble metal precursors, and the need for reproducible, large-scale processing techniques.

The journey toward industrialization begins with scalable and robust synthesis. Conventional MOF growth conditions (e.g., high temperature, polar solvents) often compromise plasmonic NP stability, leading to irreversible aggregation or degradation [46]. Benchmark systems have addressed this to some extent: Zr-based MOFs (UiO–66/67) and ZIFs (ZIF–8/67) stand out due to their rigid frameworks and superior chemical/thermal stability [134]. Room-temperature syntheses of Zr–MOFs mitigate processing-induced NP degradation, while surfactant-mediated growth (e.g., PVP, CTAB) and sacrificial template strategies (e.g., Cu_2_O, SiO_2_) enable precise structural control. Nevertheless, extending these strategies to diverse MOF families with tailored pore geometries and, crucially, ensuring reproducibility at scale remain unresolved [135,136]. Homogeneous MOF nucleation still competes with heterogeneous growth on NPs, reducing yield [137]. Systematic parametric studies on interfacial engineering are urgently needed to master heterogeneous nucleation kinetics for high-volume production.

From a practical review, precursor expenses pose a major barrier. Benchmark NP–MOFs predominantly rely on noble metal NPs (Au, Pd, Pt), whose high cost limits scalability [138]. Recent progress follows two key directions: architectural innovation to improve noble metal utilization, and exploration of alternative materials, such as low-cost transition metals (e.g., Cu, Al). However, the latter often involves a trade-off, as alternatives may exhibit inferior plasmonic performance or reduced chemical stability. A rigorous techno-economic assessment must, therefore, consider not only the upfront precursor cost but also the lifetime, activity, and recyclability of the resulting hybrid material. For MOF components, benchmark systems like ZIFs and UiO series benefit from low-cost organic linkers, enhancing their commercial viability compared to MOFs requiring rare or complex ligands [139,140].

Addressing the aforementioned challenges in a rational and efficient manner necessitates the integration of computational and data-driven approaches throughout the entire development cycle of NP–MOF hybrid materials. This approach spans key stages from fundamental property prediction to final material design. First, multiscale theoretical modeling serves as the cornerstone for understanding and predicting material behaviors. Density Functional Theory (DFT) calculations provide a theoretical basis for rational design by precisely evaluating host–guest interactions within MOF pores, interfacial charge transfer dynamics, and the electronic structure of plasmonic NPs [141,142], while the Finite–Difference Time–Domain (FDTD) method simulates EM field distributions at the nanoscale, visually predicting and optimizing the enhancement of plasmonic “hot spots” for sensing and catalysis [143]. Second, high-throughput virtual screening coupled with ML dramatically accelerates the discovery of candidate materials. By automatically extracting descriptors from large databases, ML models can rapidly predict MOF properties such as adsorption capacity and stability with high predictive accuracy, far surpassing traditional trial-and-error efficiency [144,145]. More importantly, the field is moving towards inverse design. Promising generative AI models enable researchers to define target properties, upon which the models can propose entirely new NP–MOF topological structures, thereby expanding the design space beyond human intuition. Ultimately, achieving data-driven closed-loop design represents the key future opportunity. This vision depends on establishing standardized, open-access databases and integrating them with automated experimental platforms [146,147]. Through iterative cycles of prediction and validation, the system can autonomously optimize synthesis parameters and refine designs. This intelligent paradigm is not merely a tool for acceleration, but a transformative methodology poised to directly address the core industrial challenges of scalability, cost, and stability outlined in this review [148].

In sensing applications, despite improved sensitivity through MOF-mediated preconcentration, challenges in reproducibility and selectivity persist. Suboptimal spatial arrangements of NPs in core–shell configurations weaken plasmonic coupling, while ubiquitous capping agents introduce spectral interference and obstruct molecular access to EM hotspots. To address these, future efforts should focus on developing capping-agent-free synthetic routes, designing hierarchical MOFs for improved molecular discrimination, and employing ML-enabled spectral deconvolution to resolve cross-sensitivity in complex media.

Biomedical translation faces hurdles in biocompatibility and spatiotemporal control. Unpredictable MOF degradation in physiological environments and suboptimal drug-release kinetics call for surface functionalization strategies to enhance stability and targeting specificity. Furthermore, balancing photothermal efficiency with controlled drug release under NIR irradiation is critical to minimize off-target effects [149,150]. Integrating multimodal imaging modalities will also be essential for real-time therapeutic monitoring and dosage adjustment.

In catalysis, the synergy between plasmonic excitation and MOF confinement is often hindered by insufficient mechanistic understanding. Although MOF-enabled analyte enrichment and NP-derived hot-carrier generation enhance activity, structural instability during cyclic operations and ambiguous reaction pathways impede progress [151]. Future research must prioritize stabilizing frameworks via coordination additives at metal nodes and elucidating interfacial electron transfer dynamics using in situ spectroscopic techniques.

Conclusively, NP–MOF hybrids merge plasmonic field enhancement with molecular sieving for unprecedented functionality. Their successful industrial translation hinges on four interconnected pillars derived from recent benchmark studies: mastering synthesis and interfaces through operando characterization and interfacial engineering to ensure scalable, reproducible production of stable architectures; engineering for cost and performance via architectural innovation, alternative materials exploration, and holistic techno-economic analysis; leveraging computational intelligence by employing data-driven design, high-throughput screening, and ML to accelerate discovery and optimization; and validating in application-relevant contexts by rigorously testing stability under cyclic operations, in complex media, and in prototype industrial or clinical setups.

We anticipate that continued interdisciplinary efforts—integrating materials science, chemical engineering, and data science—will drive the design of next-generation plasmonic NP–MOF hybrid platforms. These advancements will unlock their potential from fundamental research to practical applications in catalysis, biomedical theranostics, and environmental monitoring, thereby aligning with the growing commercial interest in functional nanomaterials.

## Figures and Tables

**Table 1:** Overview of the type and size distribution of MOF-encapsulated NPs.

Type of MOFs	Type of Plasmonic NPs	Size of Metal NPs	Application	Key Performance Metrics
CuNi–MOL [38]	Pd NPs	5 nm	Photocatalytic CO_2_ reduction to CO	CO selectivity ≈ 100%, yield = 48.69 μmol/g/h; 58× more active than Ni-MOL
NH_2_–UiO–66 [43]	Au NPs	15.2 ± 2.2 nm	Photocatalytic H_2_ evolution	HER rate = 664.9 μmol/g/h (28× that of CdS); AQY = 5.1% at 400 nm
ZIF–67 [44]	Au NPs	31 ± 7 nm	Photocatalytic CO_2_ reduction to CO	CO selectivity = 100%; volumetric yield 3× that of ZIF-67 powder
ZIF–67 [50]	Ag NWs	30 nm	SERS detection of thiram on fruit surfaces	LOD for thiram = 2 ng/cm^2^; linear detection on apple surface (R^2^ > 0.99)
ZIF–8 [45]	Pd NPs	30 nm	Size-selective alkene hydrogenation	100% size-selective alkene hydrogenation; stable structure and activity after cycling
ZIF–8 [54]	Pd NPs	60 nm	Gas-phase H_2_ hydrogenation	Cyclohexene hydrogenation TOF = 0.23 s^−1^; 100% size selectivity
MIL–53 [56]	Al NCs	50–150 nm	Plasmon-enhanced photocatalytic reverse water–gas shift	CO yield 3× higher than pristine Al NCs under 300 mW white light; 100% product selectivity
NU–901 [46]	Au NRs	200–350 nm	Size-selective SERS sensing of BPTCN/BPT	BPTCN signal saturation in 125 s; no response to large molecule PST-SH
ZIF–8 [61]	Au NPs	13, 34 nm	liquid-phase alkene hydrogenation	CO conversion ≈ 100% at 200 °C; 100% regioselectivity
ZIF–8 [61]	Pt NPs	2.5, 3.3, 4.1 nm	liquid-phase alkene hydrogenation	CO oxidation light-off temp = 130 °C; 1-hexene hydrogenation conversion = 7.3%

**Table 2:** Overview of the type and size distribution of NP-embedded MOF.

Type of MOFs	Type of Plasmonic NPs	Size of Metal NPs	Application	Key Performance Metrics
ZIF–8 [66]	Cu NPs	22 nm	Benzyl alcohol oxidation	Benzaldehyde yield = 66%, selectivity > 90%; stable after 5 cycles
MIL–100 [69]	Pd NPs	2.5 nm	Hydrogen storage	H_2_ uptake = 0.35 wt% at 298 K/4 MPa (1.8× that of pure MIL-100)
MOF–5 [70]	Ag NPs	/	Laser direct-writing of metallic microstructures	Laser-induced Ag^+^ reduction to form 700–800 nm metal dots; high 3D patterning precision (layer spacing = 10 μm)
MIL–101 [71]	AuNi NPs	2.0–5.0 nm	Hydrogen generation from ammonia borane hydrolysis	H_2_ production TOF = 66.2 mol_H2_·mol_cat_·min^−1^; cycle-stable for 5 runs
UiO–67 [72]	Pd@Ag NPs	2.6–3.1 nm	Selective hydrogenation of phenylacetylene	Styrene selectivity = 91% (conversion = 100%); stable structure and activity after 5 cycles
UiO–66 [73]	Au NPs	2.4 ± 0.6 nm	Plasmonic photocatalytic nitrogen fixation to NH_3_	NH_3_ yield = 18.9 mmol g_Au_^−1^h^−1^; AQE = 1.54% at 520 nm

**Table 3:** Overview of the type and size distribution of NP-decorated MOF.

Type of MOFs	Type of Plasmonic NPs	Size of Metal NPs	Application	Key Performance Metrics
HKUST–1 [74]	Ag NPs	60 nm	In situ SERS monitoring of plasmon-mediated reaction	4-NBT conversion efficiency (I_DMAB_/I_NBT_ = 1.12) > pure Ag substrate; SERS signal RSD = 5.8%
Zn–MOFs [75]	Au NSs	60 nm	Plasmon-enhanced antibacterial therapy	ROS production 2.5× that of pure Zn-MOFs; antibacterial rate > 98% against *S.aureus*/*E. coli*
UiO–66–NH2 [76]	Ag/Pd NPs	0.24 nm, 0.22 nm	Photocatalytic H_2_O_2_ and H_2_ production	H_2_ evolution rate = 448.2 μmol h^−1^; H_2_O_2_ production rate = 39.4 μmol h^−1^; stable for 4 cycles
MIL–101 [67]	Ag NPs	300 nm	SERS detection of dopamine (DA)	DA LOD = 0.32 pM (S/N = 3); recovery rate = 99.8–108.0% in human urine

## Data Availability

No new data were created.
